# Tong-Xie-Yao-Fang Regulates 5-HT Level in Diarrhea Predominant Irritable Bowel Syndrome Through Gut Microbiota Modulation

**DOI:** 10.3389/fphar.2018.01110

**Published:** 2018-09-28

**Authors:** Junchen Li, Huantian Cui, Yuzi Cai, Jin Lin, Xin Song, Zijun Zhou, Wantao Xiong, Huifang Zhou, Yuhong Bian, Li Wang

**Affiliations:** ^1^Integrative Medicine Institute, Tianjin University of Traditional Chinese Medicine, Tianjin, China; ^2^Preparation Department, Tianjin Second People’s Hospital, Tianjin, China

**Keywords:** diarrhea, irritable bowel syndrome, Tong-Xie-Yao-Fang, gut microbiota, 5-HT

## Abstract

Tong-Xie-Yao-Fang (TXYF) has been widely used for the treatment of diarrhea-predominant irritable bowel syndrome (IBS-D) in traditional Chinese medicine. However, its mechanism of action in the treatment of IBS-D remains to be fully understood. Recent reports have shown that *Clostridium* species in the gut can induce *5-HT* production in the colon, which then contributes to IBS-D. Due to the wide use of TXYF in the clinical treatment of IBS-D and the close relationship between gut microbiota and IBS-D, we hypothesize that TXYF treats IBS-D by modulating gut microbiota and regulating colonic *5-HT* levels. In this study, variation analysis of 16S rRNA was conducted to evaluate changes in the distribution of gut microbiota in IBS-D model rats after TXYF treatment. Moreover, we investigated whether TXYF could affect colonic *5-HT* levels in IBS-D model rats. We then performed fecal transplantation experiments to confirm the effects of TXYF on gut microbiota and *5-HT* levels. We found that TXYF treatment can ameliorate IBS-D and regulate *5-HT* levels in colon tissue homogenates. TXYF treatment also affected the diversity of gut microbiota and altered the relative abundance of *Akkermansia* and *Clostridium sensu stricto 1* in gut flora populations. Finally, we showed that fecal transplantation from TXYF-treated rats could relieve IBS-D and regulate *5-HT* levels in colon tissue homogenates. In conclusion, the present study demonstrates that TXYF treatment diminishes colonic *5-HT* levels and alleviates the symptoms of IBS-D by favorably affecting microbiota levels in gut flora communities.

## Introduction

Irritable bowel syndrome (IBS) is a type of functional gastrointestinal disorder associated with recurrent abdominal pain and disordered bowel habits. Epidemiological studies have shown that 5% of people in Asia and 10–15% of people in Europe and South America suffer from IBS. Moreover, the morbidity of IBS is increasing (Saito and Schoenfeld, 2002).

To date, the mechanism of IBS is not fully understood. Intestinal motility dysfunction, visceral hypersensitivity, intestinal immune activation, and gut bacteria disorder are closely related to IBS (Dupont, 2014). Enterochromaffin cells (ECs) play an important role in the occurrence of IBS. Studies have shown that there are more ECs in IBS patients, and these cells produce high levels of serotonin (*5-HT*) (Lee et al., 2010). It has been suggested that *5-HT* increases visceral sensitivity and triggers diarrhea, which leads to diarrhea-predominant IBS (IBS-D) (Atkinson et al., 2003).

Traditional Chinese medicine (TCM) is widely used in the treatment of IBS-D. Accumulated studies indicate a crucial role for gut microbiota in TCM treatment of IBS-D. For example, radix sileris can treat post-infectious IBS by modulating gut microbiota and inhibiting the serine protease pathway (Qi et al., 2015). It was previously shown that the Chinese drug “Shen Qu” could increase the abundance of *Lactobacillus* and *Bifidobacterium* and decrease the abundance of *Escherichia coli* in IBS-D patients (Zhuang et al., 2005).

Tong-Xie-Yao-Fang (TXYF) was first described in “Danxi’s Mastery of Medicine” and includes pericarpium citri reticulatae, rhizoma atractylodis macrocephalae, radix paeoniae alba, and radix sileris. TXYF has been widely used for the clinical treatment of IBS-D (Zhang et al., 2008; Huang and Zhang, 2012); however, its mechanism of action remains to be determined.

Recent studies have demonstrated that gut microbiota can influence ECs and thus affect *5-HT* levels in the gut. Serum levels of *5-HT* in germ-free (GF) animals were significantly lower than those in specific-pathogen-free (SPF) animals, and the ECs in GF and SPF animals had different morphologies (Uribe et al., 1994; Wikoff et al., 2009; Sjögren et al., 2012). Moreover, metabolites from *Clostridium* were found to up-regulate tryptophan hydroxylase (Tph) gene expression in ECs to promote *5-HT* production (Yano et al., 2015).

Based on the close relationship between gut microbiota and IBS-D, we hypothesize that TXYF treatment modulates gut microbiota and regulates gut *5-HT* levels, which alleviates the symptoms of IBS-D.

In this study, we conducted 16S rRNA variation analysis to observe the changes in gut microbiota communities in IBS-D model rats after TXYF treatment. Moreover, we investigated whether TXYF could affect *5-HT*-associated pathway gene expression in IBS-D model rats. Fecal transplantation was then used to confirm the effects of TXYF on gut microbiota and *5-HT* levels.

## Materials and Methods

### Reagents

Total DNA and RNA extraction kits, first-stand cDNA reverse transcription kits, polymerase chain reaction (PCR) kits, and primers were obtained from TianGen Biotechnology Co., Ltd., (Beijing, China). The *5-HT* ELISA test kit was obtained from Multi Science Biotechnology Co., Ltd., (Hangzhou, China).

### Preparation of TXYF

TXYF granules were provided by the First Affiliated Hospital of Tianjin University of TCM (Tianjin, China). Each TXYF granule contained the same amount of each ingredient in the TXYF formula: 45 g of pericarpium citri reticulatae, 90 g rhizoma atractylodis macrocephalae, 60 g radix paeoniae alba, and 30 g radix sileris.

### Animals

Male wistar rats, weighing 190–210 g, were purchased from Beijing Huafukang Animal Company. Rats were supplied by experimental protocols in accordance with National Institutes of Health regulations and approved by the Institutional Animal Care and Use Committee in Tianjin University of TCM. Throughout the acclimatization and study periods, all animals had access to food and water *ad libitum* and were maintained on a 12 h light/dark cycle (21 ± 2°C with a relative humidity of 45 ± 10%). Rats were housed in SPF conditions.

### Induced IBS-D Rat Model

The rat IBS-D model was induced through tail clamping and restraint stress according to an established protocol (Katz et al., 1981). Briefly, rats were provoked with a clamp on the 1/3 proximal portion of the tail for 30 min. The rats were then anesthetized with chloral hydrate, and the upper limbs and chest were restrained with packaging tape. The restraint lasted 2 h following recovery from anesthetization. To establish the IBS-D rat model, the rats underwent tail clamping and restraint once per day for 14 days followed by 14 days of rest. To verify the IBS-D rat model, visceral hypersensitivity was measured using the abdominal withdrawal reflex (AWR) scoring system (**Supplementary Material**) as described previously (Yu et al., 2018), and the number of fecal pellets was used to evaluate the severity of diarrhea in each group as described previously (Liang et al., 2012). In brief, each rat was housed separately in a metal cage and papers were placed under each cage. After 2 h, the numbers of fecal pellets that dropped onto the paper were counted.

### Groups

For the TXYF treatment experiment, rats were randomly divided into three groups (*n* = 10 per group): Control group, IBS group, and TXYF + IBS group. Rats in the IBS and TXYF + IBS groups received tail clamping and restraint stress to induce IBS-D. Rats in the TXYF + IBS group were treated orally with 2 mL TXYF (19.2 g/kg rat weight) once per day, whereas rats in the Control and IBS groups were treated orally with 2 mL normal saline per day.

Fecal transplantation experiments were performed based on an established protocol (Borody et al., 2013). In brief, donor rats were randomly divided into four groups (*n* = 6 per group): Control, TXYF, IBS and TXYF + IBS. Normal feed was given to the Control group, and the TXYF group received 2 ml of TXYF (19.2 g/kg rat weight) orally per day. The IBS and TXYF + IBS groups received tail clamping and restraint stress to induce IBS-D. The TXYF + IBS group received 2 mL of TXYF (19.2g/kg rat weight) orally per day, whereas the IBS group received 2 mL of saline per day. After 28 days, stools from each group of donor rats were collected daily for the subsequent 28 days under sterile conditions in a laminar flow hood. Stools from donor rats in each group were pooled, and 100 mg of the pooled stool samples were resuspended in 1 ml of sterile normal saline. The solution was vigorously mixed for 10 s using a bench-top vortex mixer followed by centrifugation at 800 g for 3 min. The supernatant was collected and used as transplant material as described below. Fresh transplant material was prepared on the day of transplantation 10 min prior to oral gavage to prevent changes in bacterial composition. Recipient rats were randomly divided into four groups (*n* = 8 per group). Each group of recipient rats received tail clamping and restraint stress to induce IBS-D, and received fresh transplant material (1 ml for each rat) orally from one of four donor groups per day for 28 days.

### Histology

Rat colons were removed and fixed in paraformaldehyde. The colon was then embedded in paraffin and subsequently cut into 5 μm sections. Sections were stained with hematoxylin and eosin (H&E).

### Fecal 16S rRNA Sequencing

After TXYF treatment, feces from the Control, IBS, and TXYF + IBS groups were simultaneously obtained under sterile conditions in a laminar flow hood. Total DNA was extracted from fecal samples using the CTAB/SDS method. DNA quantity and purity were evaluated on 1% agarose gels. DNA was diluted to 1 ng/μL with sterile water. The diluted DNA from each sample was used as the template to amplify the V4 region of 16S rRNA (16SV4) with specific barcoded primers (515F: GTGCCAGCMGCCGCGGTAA 806R: GGACTACHVGGGTWTCTAAT). All PCR reactions were carried out with Phusion^®^High-Fidelity PCR Master Mix (New England Biolabs). The PCR products were mixed with an equal volume of 1× loading buffer containing SYBR green and visualized after electrophoresis on a 2% agarose gel. Samples with a bright band between 400 and 450 bp were chosen for further experiments. The PCR products were then mixed in equidensity ratios and purified with the GeneJETTM Gel Extraction Kit (Qiagen, Germany). The TruSeq^®^DNA PCR-Free Sample Preparation Kit (Illumina, United States) was used to generate sequencing libraries following the manufacturer’s protocols, and index codes were added. The library quality was assessed using a Qubit@ 2.0 Fluorometer (Thermo Scientific) and an Agilent Bioanalyzer 2100 system. The library was sequenced on the IlluminaHiSeq2500 platform, and 250 bp paired-end reads were generated. Paired-end reads were assigned to samples based on their unique barcode and truncated by trimming the barcode and primer sequences. The sequences were then merged using FLASH (V1.2.7) to generate raw tags (Mago and Salzberg, 2011). Raw tags were rarified using QIIME(V1.7.0) quality controlled process (Caporaso et al., 2010). Sequences that contain more N-terminals or contain more inferior quality bases were filtered out(Bokulich et al., 2013). There are two main steps for filtration: (a) Tags interception: cut raw tags from a continuous low-quality value (default mass threshold < = 19) base to the first low quality base point of the set length (default length value 3); and (b) Tags Length Filter: further filter out tags with continuous high-quality bases of less than 75% of tag length. After filtration, clean tags were obtained. Clean tags were compared against the reference database (Gold) using a UCHIME algorithm to detect chimeric sequences (Edgar et al., 2011). The chimeric sequences were then removed (Haas et al., 2011) to obtain the effective tags.

### OTU Clustering and Species Annotation

The obtained effective tags were clustered using Uparse software(Edgar, 2013). Sequences with ≥97% similarity were assigned to the same OTUs. Representative sequences for each OTU was selected for further annotation. For each representative sequence, the mothur algorithm(Wang et al., 2007) in the SSUrRNA database(Quast et al., 2013) was used to obtain taxonomic information. Multiple sequence alignments were conducted using MUSCLE software(Edgar, 2004) to study the phylogenetic relationship of different OTUs and the differences between the dominant species in different samples (groups). Finally, OTU abundance was normalized to the number of sequences in the sample with the fewest sequences. Subsequent analyses of alpha diversity and beta diversity were performed using the normalized data.

### Cytokine Quantification by Enzyme-Linked Immunoassay (ELISA)

Colon tissue (0.1 g) was weighed and homogenized in 900 μl normal saline by ultrasonic trituration followed by centrifugation at 3000 rpm for 15 min to obtain the colon tissue homogenate. The levels of 5-HT in the colon tissue homogenate were determined by ELISA analysis according to the manufacturer’s instructions (Multi Science Biotechnology Co., China).

### RNA Isolation and Real-time Reverse Transcription Quantitative Polymerase Chain Reaction

Total RNA isolation from rat colons and colon mucosa and cDNA synthesis were performed using kits(TianGen Biotechnology Company). According to the manufacturer’s instructions Quantitative RT-PCR (qRT-PCR) was used to evaluate the mRNA expression levels of Serotonin reuptake transporter (*Sert*) and Serotonin 2A receptor (*5Ht2ar*) in the colon and *Tph1* and *Tph2* in colon mucosa as previously described (Citri et al., 2011). All samples were run in triplicate using a BioRad iQ5 qPCR machine. *β-actin* was used as a loading control. Quantification was done using the 2[-Delta Delta C(T)] method (Livak and Schmittgen, 2001). The sequences of all primers are listed in **Table 1**.

**Table 1:** Primer sequences for rat target genes.

Genes	Primer sequence (5′–3′)
*β-actin*	Forward: ACC GTG AAA AGA TGA CCC AGA T
	Reverse: CCA GAG GCA TAC AGG GAC AA
*Ser*t	Forward: ATC TCC TAG AAC CCT GTA AC
	Reverse: GAA ATG GAC CTG GAG TAT TG
*Tph1*	Forward: CAC TCA CTG TCT CTG AAA ACG C
	Reverse: AGC CAT GAA TTT GAG AGG GAG G
*Tph2*	Forward: TAA ATA CTG GGC CAG GAG AGG
	Reverse: GAA GTG TCT TTG CCG CTT CTC
*5Ht2ar*	Forward: CCC TGC TCA ATG TGT TTG TC
	Reverse: ACT GTC TGC TCA GCA TCT TC


### Statistics

All data are reported as the mean ± standard deviation (mean ± SD) for independent experiments. Statistical differences between the experimental groups were examined by analysis of variance (ANOVA) followed by Newman–Keuls multiple comparison test using SPSS version 20.0. A *P*-value < 0.05 was considered statistically significant. Curve-fitting was carried out using the graphical package GraphPad Prism5.

## Results

### Effects of TXYF on IBS-D Rats

**FIGURE 1:**
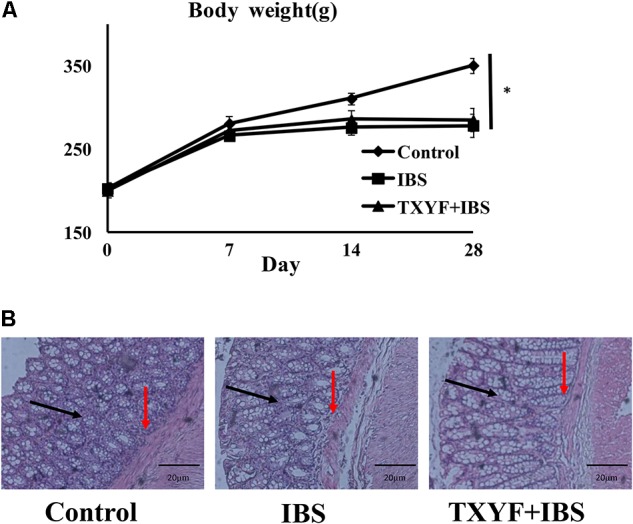
Changes in IBS-D model rat body weight and colonic histology after TXYF treatment. **(A)** Body weight in the IBS group was lower than that in the Control group, and TXYF treatment did not increase the body weight of IBS-D model rats. **(B)** H&E staining revealed no pathological changes in each group. Arrows in black indicate mucosas, and arrows in red indicate submucosas. Magnification: 20×. Control; IBS; and TXYF+IBS (*n* = 10 per group). Data are presented as mean ± SD. ^∗^*p* < 0.05 and ^∗∗^*p* < 0.01.

Body weight and fecal pellets were observed to assess the induction of IBS-D in rats. In addition, the colons of rats were stained with H&E to observe histological changes in each group. The initial body weight (201.6 ± 7.8 g vs. 202.3 ± 6.4 g vs. 199.8 ± 8.0 g, *P* > 0.05, **Figure 1A**) and number fecal pellets were not statistically different between groups (1.7 ± 0.8 vs. 1.6 ± 0.6 vs. 1.8 ± 0.9, *P* > 0.05, **Table 2**). After IBS-D induction and TXYF treatment, the body weight in the IBS group was significantly lower than that in the Control group (278.3 ± 13.7 g vs. 350.7 ± 9.0 g, *P* < 0.05, **Figure 1A**), and there were no significant differences in body weight between the TXYF + IBS and IBS groups (284.8 ± 14.0 g vs. 278.3 ± 13.7 g, *P* > 0.05, **Figure 1A**). Moreover, the IBS group produced significantly more fecal pellets than the Control group (3.9 ± 0.6 vs. 1.7 ± 1.0, *P* < 0.01 **Table 2**), whereas the TXYF + IBS group produced fewer fecal pellets than the IBS group (2.1 ± 1.6 vs. 3.9 ± 0.6, *P* < 0.05, **Table 2**). H&E staining showed that the colonic epithelial cells in each group were arranged regularly. Moreover, no pathological changes, such as cell inflammation or colonic epithelial cell damage, were observed in any group (**Figure 1B**).

**Table 2:** Number of fecal pellets from IBS-D rats before and after TXYF treatment.

Group	Numbers of fecal pellets
**Number of fecal pellets before TXYF treatment.**	
Control	1.7 ± 0.8
IBS	1.6 ± 0.6
TXYF + IBS	1.8 ± 0.9
**Number of fecal pellets after TXYF treatment.**	
Control	1.7 ± 1.0
IBS	3.9 ± 0.6 ##
TXYF + IBS	2.1 ± 1.6 ^∗^


*Control; IBS; and TXYF+IBS (n = 10 per group). Data are presented as mean ± SD. ^#^*P* < 0.05 compared with the Control group, ^##^*P* < 0.01 compared with the Control group, ^∗^*P* < 0.05 compared with the IBS group, ^∗∗^*P* < 0.01 compared with the IBS group.*

### TXYF Influences the Gut Microbiota Community in IBS-D Rats

**FIGURE 2:**
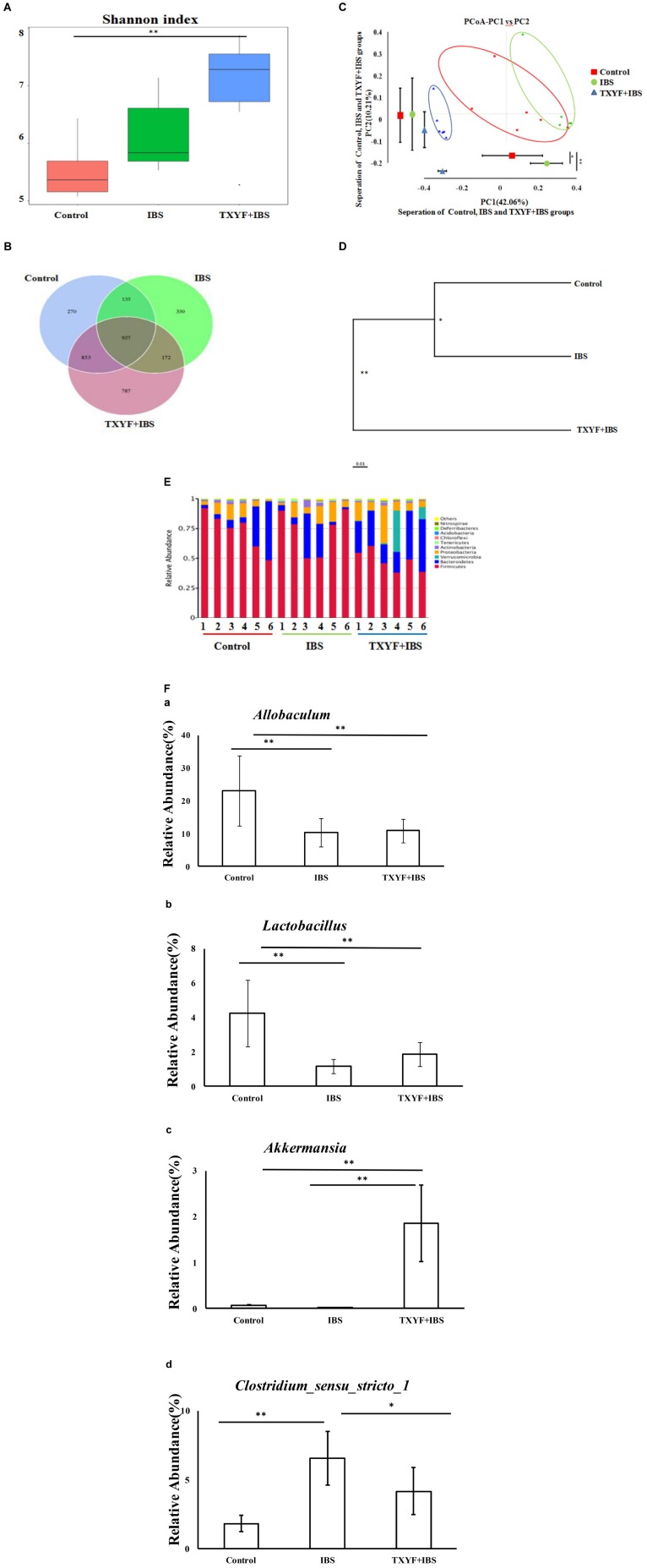
TXYF influences the gut microbiota of IBS-D rats. **(A)** Shannon indexes calculated after rarefying to an equal number of sequence reads for all samples. The Shannon indexes were higher in the IBS and TXYF + IBS groups than in the Control group; **(B)** Venn diagram indicating the different numbers of OTUs in each group; **(C)** PCoA score based on weighted Unifrac metrics was different in each group; **(D)** System clustering tree of gut microbiota based on weighted Unifrac metrics; **(E)** TXYF treatment changed the microbial community at the phylum level (bar plot); and **(F)** At the genus level, the relative abundances of *Allobaculum* **(a)** and *Lactobacillus* **(b)** were lower in the IBS-model group than in the Control group. TXYF treatment remarkably increased the relative abundance of *Akkermansia* **(c)** compared with both the IBS-model group and the Control group. Moreover, the relative abundance of *Clostridium sensu stricto 1* **(d)** was higher in the IBS-model group than in the Control group. TXYF treatment significantly decreased the abundance of *Clostridium sensu stricto 1* in the gut. Control; IBS; and TXYF+IBS (*n* = 6 per group). Data are presented as mean ± SD. ^∗^*P* < 0.05 and ^∗∗^*P* < 0.01.

High-throughput sequencing of 16S rRNA was done to study the changes in gut microbiota in IBS-D model rats after TXYF treatment. We obtained 1,435,256 usable reads and 3,504 operational taxonomic units (OTUs) from 18 samples. The Shannon Diversity index was higher in the IBS and TXYF + IBS groups than in the Control group (**Figure 2A**). Moreover, Venn diagram analysis revealed that 957 OTUs were common to all three groups, 1,092 OTUs were present in both the Control and IBS groups, 1,810 OTUs were present in both the Control and TXYF + IBS groups, and 1,129 OTUs were present in both the IBS and TXYF + IBS groups (**Figure 2B**). Principal coordinates analysis (PCoA) showed that gut microbiota in the IBS and TXYF + IBS groups were statistically different from the microbiota in the Control group (**Figure 2C**). In agreement with this result, system clustering trees indicated significant differences between groups (**Figure 2D**).

We further investigated the gut microbiota species and their relative abundance. At the phylum level, 10 phyla could be found in all samples, and the most abundant phyla in all samples were *Bacteroidetes* and *Firmicutes* (**Figure 2E**). The relative abundances of *Firmicutes* and *Bacteroidetes* were significantly higher and lower, respectively, in the IBD model groups than in the Control group. TXYF treatment increased the relative abundance of *Bacteroidetes* and decreased the relative abundance of *Firmicutes*, as compared with the Control group (**Figure 2E**). At the genus level, the relative abundances of *Allobaculum* and *Lactobacillus* were lower in the IBS-model group than in the Control group, whereas TXYF treatment did not influence the relative abundances of *Allobaculum* and *Lactobacillus* in IBS model rats (**Figure 2F**). TXYF treatment remarkably increased the relative abundance of *Akkermansia* compared with both the IBS-model and Control groups (**Figure 2F**). Moreover, the relative abundance of *Clostridium sensu stricto 1* was higher in the IBS-model groups compared with the Control group, and TXYF treatment significantly decreased the abundance of *Clostridium sensu stricto 1* in the gut (**Figure 2F**). Taken together, these results demonstrate that TXYF can modulate gut microbiota composition in IBS-D model rats.

### Level of *5-HT* and Expression of Associated Pathway Genes in IBS-D Rats Treated With TXYF

**FIGURE 3:**
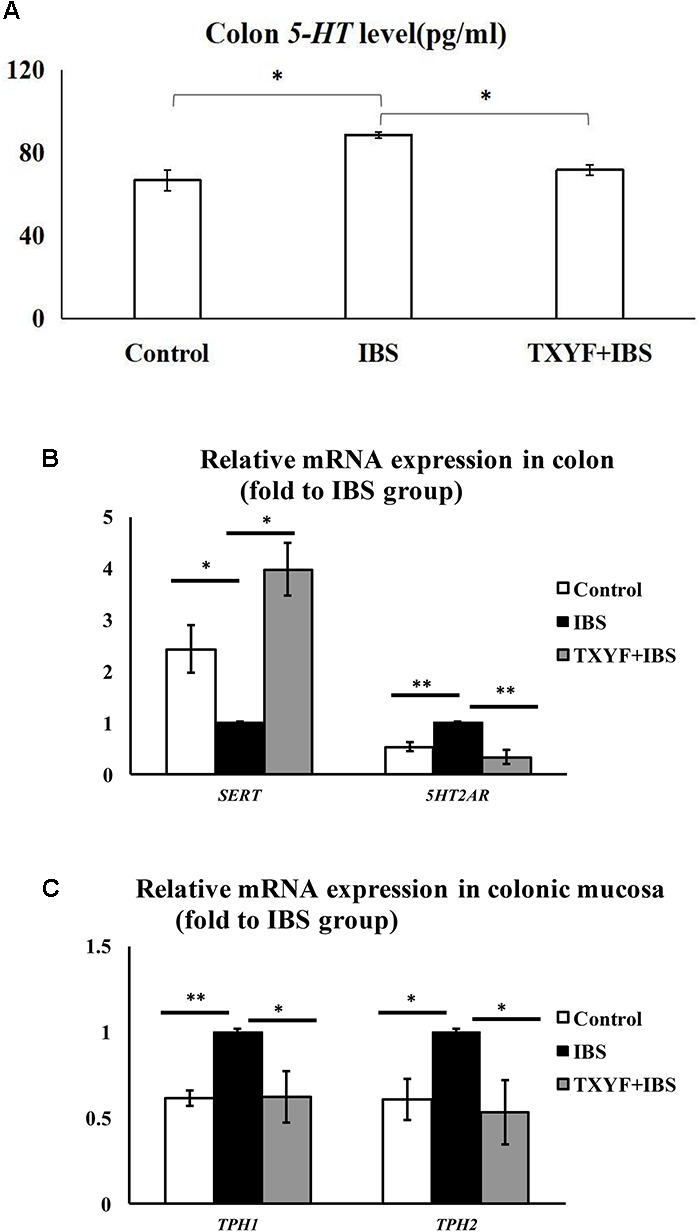
TXYF treatment affected the level of *5-HT* and the expression of associated pathway genes in IBS-D rats. **(A)** TXYF treatment decreased the colonic *5-HT* level in IBS-D model rats. **(B)** TXYF treatment up-regulated the expression of *Sert* and down-regulated the expression of *5Ht2ar* in colonic tissue of IBS rats (Fold change relative to the IBS group is shown). **(C)** After TXYF treatment, the mRNA expression levels of *Tph1* and *Tph2* in colonic mucosa of IBS rats were down-regulated (Fold change relative to the IBS group is shown). Control; IBS; and TXYF+IBS (*n* = 10 per group). Data are presented as mean ± SD. ^∗^*P* < 0.05 and ^∗∗^*P* < 0.01.

The level of 5-HT in colon tissue homogenate was investigated using ELISA. The level of *5-HT* was higher in the IBS group than in the Control group (88.72 ± 1.52 pg/ml vs. 66.77 ± 5.16 pg/ml, *P* < 0.05, **Figure 3A**). TXYF treatment decreased the level of *5-HT* in colonic tissue homogenate compared with the IBS group (71.80 ± 2.39 pg/ml vs. 88.72 ± 1.52 pg/ml, *P* < 0.05, **Figure 3A**). We also investigated the expression of four genes related to the *5-HT* pathway: *Sert* (involved in *5-HT* transport), *5Ht2ar* (a receptor for *5-HT*), and *Tph1* and *Tph2* (associated with *5-HT* synthesis). The expression of these genes in colonic mucosa was evaluated using qPCR. Colonic *5Ht2ar* gene expression was higher and colonic *Sert* gene expression was lower in the IBS group compared with the Control group (*P* < 0.01 and *P* < 0.05, respectively, **Figure 3B**), whereas TXYF treatment up-regulated *Sert* gene expression and down-regulated *5Ht2ar* gene expression compared with the IBS group (*P* < 0.01 and *P* < 0.05, respectively, **Figure 3B**). *Tph1* and *Tph2* gene expression in colonic mucosa was higher in the IBS group than in the Control group (*P* < 0.01 and *P* < 0.05, respectively, **Figure 3C**), whereas TXYF treatment down-regulated *Tph1* and *Tph2* gene expression compared with the IBS group (*P* < 0.05 for both genes, **Figure 3C**).

### Effects of TXYF Fecal Transplantation on IBS-D Rats

**FIGURE 4:**
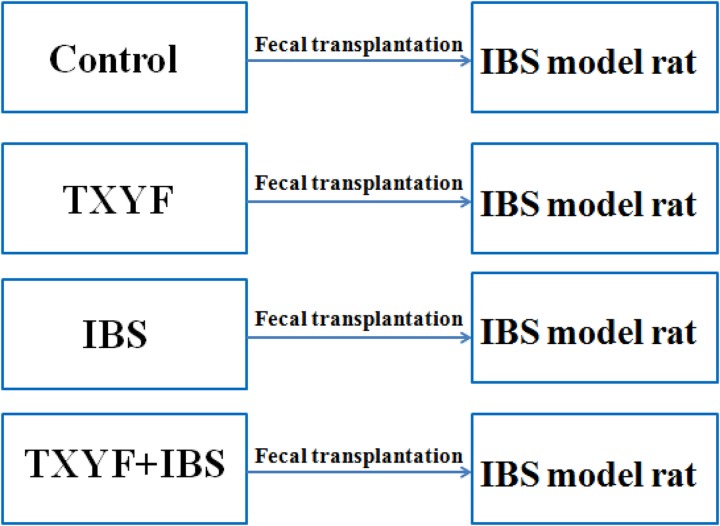
Groups in the fecal transplantation experiment. Fecal samples from Control, TXYF, IBS, and TXYF + IBS rats were transplanted to IBS model rats.

**FIGURE 5:**
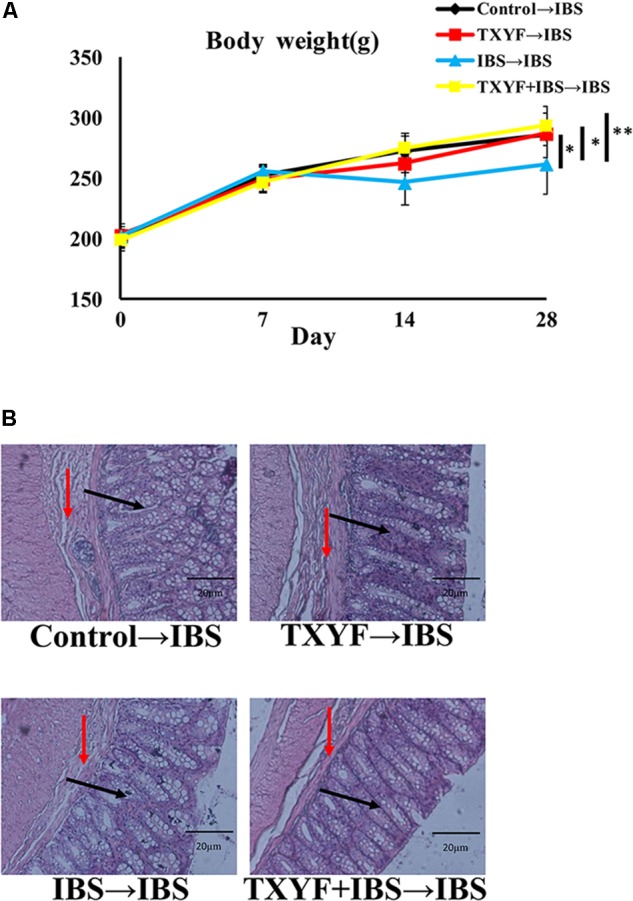
Changes in body weight and colonic histology after fecal transplantation. **(A)** Body weight of rats in the IBS→IBS fecal transplantation group was lower compared with the Control→IBS, TXYF→IBS, and TXYF + IBS→IBS fecal transplantation groups. **(B)** H&E staining revealed no pathological changes in each group. Arrows in black indicate mucosas, and arrows in red indicate submucosas. Magnification: 20×. Control→IBS; TXYF→IBS; IBS→IBS; and TXYF + IBS→IBS (*n* = 8 per group). Data are presented as mean ± SD. ^∗^*P* < 0.05 and ^∗∗^*P* < 0.01.

**FIGURE 6:**
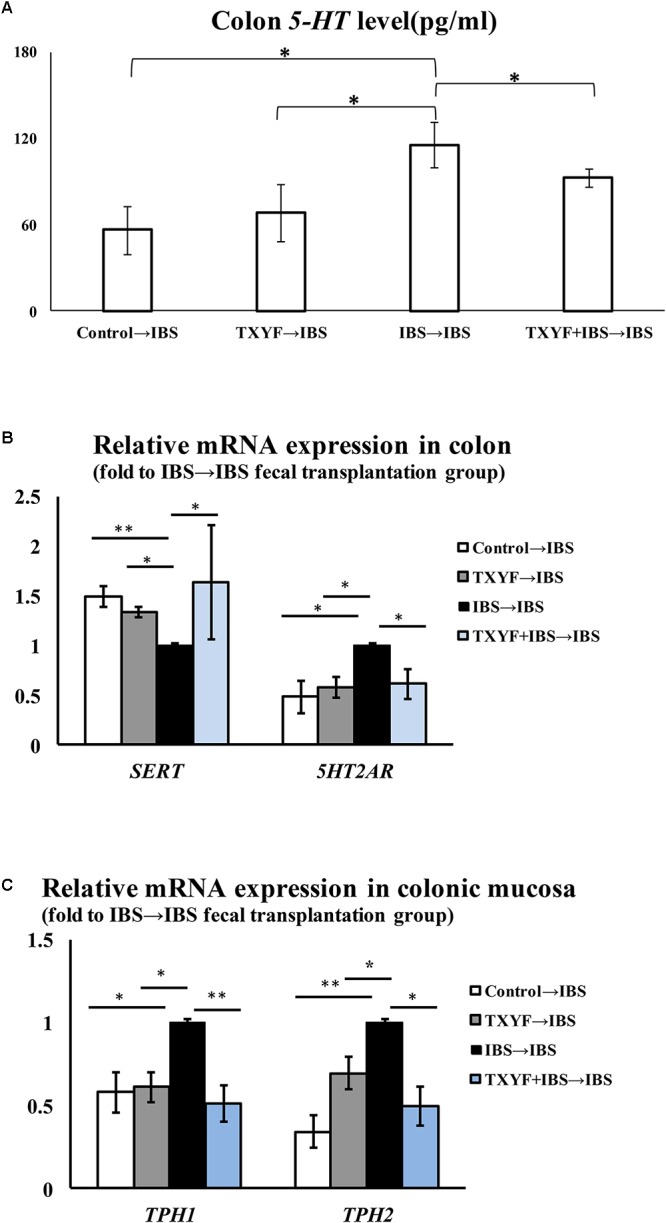
Level of *5-HT* and associated pathway genes after fecal transplantation. **(A)** The level of *5-HT* in TXYF→IBS colonic homogenates was lower than that in IBS→IBS colonic homogenates. **(B)** The expression of *Sert* in the colon was up-regulated in the Control→IBS, TXYF→IBS, and TXYF + IBS→IBS fecal transplantation groups compared with the IBS→IBS fecal transplantation group, whereas the expression of *5Ht2ar* in the colon was down-regulated in the Control→IBS, TXYF→IBS and TXYF + IBS→IBS fecal transplantation groups compared with the IBS→IBS fecal transplantation group (Fold change relative to the IBS→IBS group is shown). **(C)** The expression of *Tph1* and *Tph2* in colonic mucosa was down-regulated in the Control→IBS, TXYF→IBS, and TXYF+IBS→IBS fecal transplantation groups compared with the IBS→IBS fecal transplantation group (Fold change relative to the IBS→IBS group is shown). Control→IBS; TXYF→IBS; IBS→IBS; and TXYF+IBS→IBS (*n* = 8 per group); Data are presented as mean ± SD. ^∗^*P* < 0.05 and ^∗∗^*P* < 0.01.

Accumulated clinical studies have shown that fecal microbiota transplantation alleviates IBS-D by modulating the gut microenvironment (Hua et al., 2017; Juncadella and Moss, 2017; Elsalhy and Mazzawi, 2018). To further confirm that TXYF affects gut *5-HT* levels in IBS-D model rats by regulating gut microbiota, the gut microbiota of the Control, TXYF, IBS-D and IBS-D + TXYF rats were transplanted to IBS-D rats (**Figure 4**). After fecal transplantation, the body weights in the IBS→IBS group were significantly lower than those in the Control→IBS (261.4 ± 24.0 g vs. 286.0 ± 18.3 g, *P* < 0.05, **Figure 5A**), TXYF→IBS (261.4 ± 24.0 g vs. 286.7 ± 4.7 g, *P* < 0.05, **Figure 5A**), and TXYF+IBS→IBS groups (261.4 ± 24.0 g vs. 293.3 ± 16.5 g, *P* < 0.01, **Figure 5A**). Moreover, there were significantly more fecal pellets produced by the IBS→IBS group than by the Control→IBS (7.4 ± 2.7 vs. 4.0 ± 0.8, *P* < 0.05, **Table 3**) and TXYF + IBS→IBS groups (7.4 ± 2.7 vs. 5.6 ± 0.7, *P* < 0.05, **Table 3**). H&E staining revealed no colonic histological changes in any of the groups (**Figure 5B**). *5-HT* levels in colonic tissue homogenate were higher in the IBS→IBS group than in the Control→IBS (115.62 ± 15.49 pg/ml vs. 56.48 ± 16.58 pg/ml, *P* < 0.05, **Figure 6A**), TXYF→IBS (115.62 ± 15.49 pg/ml vs. 68.26 ± 19.73 pg/ml, *P* < 0.05, **Figure 6A**), and TXYF + IBS→IBS groups (115.62 ± 15.49 pg/ml vs. 92.63 ± 6.10 pg/ml, *P* < 0.05, **Figure 6A**). In addition, colonic *5Ht2ar* gene expression was up-regulated in the IBS→IBS group compared with the Control→IBS, TXYF→IBS and TXYF + IBS→IBS groups (*P* < 0.05, **Figure 6B**), whereas *Sert* gene expression in the colon was down-regulated in the IBS→IBS group compared with the Control→IBS, TXYF→IBS and TXYF + IBS→IBS groups (*P* < 0.01, *P* < 0.05, and *P* < 0.05, respectively, **Figure 6B**). *Tph1* gene expression in colonic mucosa was higher in the IBS→IBS group than in the Control→IBS, TXYF→IBS and TXYF + IBS→IBS groups (*P* < 0.05, *P* < 0.05, and *P* < 0.01, respectively, **Figure 6C**). *Tph2* gene expression in colonic mucosa was up-regulated in the IBS→IBS group compared with the Control→IBS, TXYF→IBS and TXYF + IBS→IBS groups (*P* < 0.01, *P* < 0.05, and *P* < 0.05, respectively, **Figure 6C**).

**Table 3:** Number of fecal pellets from IBS-D rats before and after fecal transplantation.

Group	Numbers of
	fecal pellets
**Number of fecal pellets before fecal transplantation.**	
Control→IBS	1.6 ± 0.9
TXYF→IBS	1.7 ± 0.6
IBS→IBS	1.9 ± 0.9
TXYF + IBS→IBS	1.8 ± 0.7
**Number of fecal pellets after fecal transplantation.**	
Control→IBS	4.0 ± 0.8 ^∗^
TXYF→IBS	4.7 ± 2.6
IBS→IBS	7.4 ± 2.7
TXYF + IBS→IBS	5.6 ± 0.7 ^∗^


*Control→IBS; TXYF→IBS; IBS→IBS; TXYF+IBS→IBS (*n* = 8 per group). Data are presented as mean ± SD. ^∗^*P* < 0.05 compared with the IBS→IBS group, ^∗∗^*P* < 0.01 compared with the TXYF + IBS→IBS group.*

## Discussion

In this study, we established a rat IBS-D model through tail clamping and restraint-induced stress. After 4 weeks, the body weight of rats was lower and the number of fecal pellets was higher in the IBS-D group than in the Control group, indicating that rats incurred diarrhea. In addition, H&E staining revealed no pathological changes to colon epithelial cells in the IBS group, which is in agreement with previous studies that IBS-D is not accompanied by inflammatory responses and colonic epithelial cell damage (Brun et al., 2017). Interestingly, TXYF also decreased the number of fecal pellets produced by IBS-D model rats, indicating that TXYF could relieve diarrhea in these rats.

We also investigated the changes in microbiological composition using high-throughput sequencing. In agreement with previous work, our results demonstrate that the alpha diversity of the gut microbiota community was increased in the IBS-D model group, which had a higher Shannon Diversity index than the Control group (Labus et al., 2017). Moreover, PCoA analysis and the system clustering tree revealed significant distances between each group, indicating that the beta diversity of gut microbiota of IBS-D model rats and TXYF-treated rats differed from that of the Control rats. According to recent studies, a high ratio of *Firmicutes* to *Bacteroidetes* (F-B ratio) is closely associated with IBS (Jeffery et al., 2012; Labus et al., 2017). We also found that the F-B ratio was higher in IBS-D model rats than in control rats and that TXYF treatment could decrease the F-B ratio in IBS-D model rats. We also found that the relative abundances of *Allobaculum* and *Lactobacillus* were lower in IBS-D model rats than in control rats. *Lactobacillus* has been demonstrated to have beneficial clinical effects on IBS (Fan et al., 2006r; Hyun et al., 2008). *Allobaculum* produces short chain fatty acids that could relieve diarrhea in IBS-D patients by altering the intestinal transit time (Camilleri, 2015; Zhang et al., 2015). However, TXYF treatment did not change the relative abundance of *Allobaculum* and *Lactobacillus* in IBS-D model rats. By contrast, we observed a remarkable increase in the abundance of *Akkermansia* after TXYF treatment. Studies have shown that *Akkermansia muciniphila* is a mucin degrader and prevents obesity by interacting with intestinal epithelial cells (Derrien et al., 2008; Everard et al., 2013). However, the possible role of *A. muciniphila* in IBS still needs to be further studied. Interestingly, TXYF treatment decreased the fecal abundance of *Clostridium*, which is implicated in the stimulation of ECs to produce *5-HT*. In the gut, cholate is transformed into deoxycholate through the 7α-dehydroxylation activity of *Clostridium* (Kitahara et al., 2001; Narushima et al., 2006). Deoxycholate could increase *5-HT* synthesis in colon (Yano et al., 2015).

The modulation of gut microbiota may be attributed to an active component in TXYF. For example, radix sileris, an ingredient in TXYF, may prevent IBS by modulating gut microbiota species (Qi et al., 2015). Another TXYF ingredient, rhizoma atractylodis macrocephalae, was found to increase the abundance of *Bifidobacterium* and *Lactobacillus* in the mice gut (Yan et al., 2011). Further studies need to be carried out to determine which TXYF component influences gut microbiota species composition.

We also demonstrated that TXYF can decrease 5-HT levels in the gut. 5-HT is an important factor in regulating intestinal motility (Gershon and Tack, 2007). The excessive production of 5-HT induces high visceral sensitivity, which is an important mechanism of IBS-D (Stasi et al., 2014). Our current study also demonstrated that TXYF may decrease the level of 5-HT in colon by up-regulating colonic *Sert* expression and down-regulating colonic *Tph1*, *Tph2*, and *5Ht2ar* expression in IBS-D model rats. Most of the 5-HT in our body is synthesized by the ECs. As the rate-limiting enzyme of 5-HT biosynthesis, Tph catalyzes the transformation of tryptophan into 5-HT (Katsumata et al., 2018). *Tph1* and *Tph2* are two isoenzymes of *Tph* that exist in different cells (Walther et al., 2003). In the gut, *Tph1* is predominantly expressed in ECs whereas *Tph2* is highly expressed in enteric nerves (Li et al., 2011). *5Ht2ar* is a receptor of 5-HT. Binding of 5-HT and 5Ht2ar blocks voltage-gated K^+^ channels and increases visceral sensitivity by inducing the excitation of enteric neurons (Zhang and Arsenault, 2005). *Sert* is found on the membrane of intestinal epithelial cells and is involved in the re-uptake of 5-HT (Chumpitazi and Shulman, 2016). Excess 5-HT is transferred into epithelial cells through *Sert* where it is inactivated. Studies have shown that inhibiting *Sert* expression induces the sensitivity of primary neurons to 5-HT, which could ultimately increase visceral sensitivity (Gwee et al., 2009; Chumpitazi and Shulman, 2016).

We also performed fecal transplantation experiments to determine whether TXYF alleviates IBS-D by modulating gut bacteria. Fecal transplantation is a novel method to study the relationship between an administered treatment and gut bacterial flora. Research has shown that mice receiving fecal transplants from *Ganoderma lucidum*-treated mice have less high fat diet-induced obesity, suggesting that a key factor in the reduction of obesity by *G. lucidum* is its effect on gut bacteria (Chang et al., 2015). Our results also demonstrate that fecal samples from TXYF-treated rats can reduce IBS-D and inhibit *5-HT* levels in the gut.

In conclusion, we have demonstrated that TXYF treatment diminishes colonic *5-HT* levels and alleviates the symptoms of IBS-D by favorably affecting microbiota levels in gut flora communities.

## Author Contributions

JL and HC wrote the manuscript. YC, JL, XS, HC, and JL conducted the animal experiments. LW, ZZ, WX, and HZ finished molecular bioassay. YB provided technical guidance for the whole work. All the authors approved the final draft.

## Conflict of Interest Statement

The authors declare that the research was conducted in the absence of any commercial or financial relationships that could be construed as a potential conflict of interest.
