# Supplementary material for: Tong-Xie-Yao-Fang Regulates 5-HT Level in Diarrhea Predominant Irritable Bowel Syndrome Through Gut Microbiota Modulation
Source: Front Pharmacol. 2018 Sep 28;9:1110. doi: 10.3389/fphar.2018.01110 (PMC6172324; doi:10.3389/fphar.2018.01110)
Supplement: Supplementary file 1 [file Image_1.pdf]

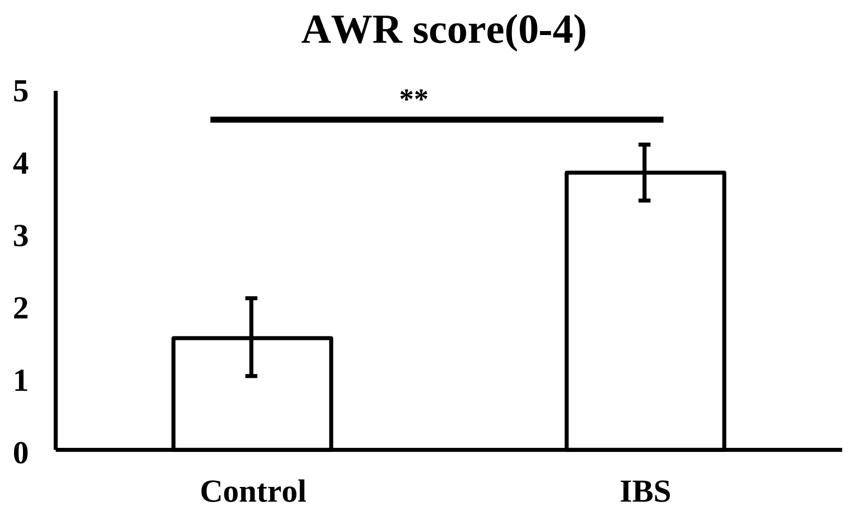

sFig. 1: The AWR score of the IBS group was significantly higher than that of the control group.

Control; IBS (n=10 per group)

Data are presented as mean ± SD. *: $p < 0.05$, **: $p < 0.01$.
